# Maladies Caused by the Air We Breathe

**Published:** 1906-03-24

**Authors:** 


### MALADIES CAUSED BY THE AIR WE BREATHE.

There is no higher function of medical policy than the application of scientific knowledge to the prevention of disease and the preservation of health. Alike to the individual and to the State, such ends rank in the first order of importance, and those who seek their promotion deserve well both of the present and future generations. An admirable presentation of work of this order is to be found in the recently published Harben Lectures for 1905.* Their author, Dr. Thomas Oliver, is well known as an authority on scientific and practical hygiene, and the present volume will certainly add to his already established reputation. It is true that much of what he has to say depends upon facts which to those actively concerned in sanitary science can hardly claim the merit of novelty. But even here the information is unusually well arranged, and is applied to practical ends with much force and ingenuity. Hence, while the volume will be welcome to medical sanitarians, it is not less suitable to the general public, and may profitably be employed in the education of those local authorities without whose cooperation the application of scientific principles to practical hygiene is impossible. Even the best and wisest general rules, however, need for their success an intelligent interpretation and comprehension on the part of the individuals immediately concerned. Thus in the last resort it is necessary to secure an educated public opinion which will recognise and condemn personal departures from sanitary law as actions essentially anti-social in character and opposed to the public interest. Dr. Oliver's lectures certainly tend to promote these highly desirable ends, and therefore deserve wide and generous recognition. To say that bodily health and activity are conditioned by the quality of the atmosphere introduced through the lungs into the blood is a trite remark, but it is none the less useful to study the extent of the application of this doctrine. Possibly the general attention is mainly directed to the grosser contaminations due to the introduction of poisonous fumes, smoke, and other by-products of industrial activity into the atmosphere, though the general indignation also recognises more or less fitfully, however, the inconveniences which arise from the imperfect ventilation of public rooms and conveyances. The more subtle dangers which may lurk in the atmosphere, and the responsibility of the individual for many of these, receive a much slighter measure of attention. Yet in all probability it is from these directions that the more widespread evils spring. The degree of danger in an imperfectly renewed atmosphere in human habitations is not to be measured merely, or even mainly, by an increased percentage of carbonic acid gas. Inimical as this product of animal respiration undoubtedly is, there are yet in air that has been breathed substances much more prejudicial to health and vigour, and it is to these organic poisons that the ill results of imperfect ventilation are mainly due. The direct effects of these substances are bad enough in all conscience, but their indirect consequences are still more disastrous. It is, to mention but a single instance, impure air that paves the way for the operation of the tubercle bacillus, and thus is found the main explanation of the fact that "the mortality from tubercular phthisis is in direct proportion to the density of the population, no matter in what town or country the observations are made." No one will deny that existing social conditions which are beyond the limits of individual, and perhaps even of corporate, reform tend directly to the promotion of these results. All the same, they can be modified in some degree by personal activity and by a quickening of the public conscience; and in aiding these methods the medical profession is rendering worthy social and imperial service. It is, of course, well known that the tubercle bacillus is largely disseminated by dust which has arisen from the dried expectoration of tuberculous patients, and that were attempts to check this successful, the disease would largely disappear. But it is hardly less important—and indeed in the meantime it may be of more immediate service—for the public to know that the ability to resist the inroads of pathogenic organisms is promoted by the breathing of pure air, and that many diseases which depend immediately upon a specific cause would be avoided by the existence of that bodily vigour which is prejudiced by the systematic use of ill-ventilated rooms. Diseases due to the air we breathe are in part, it must be allowed, and in existing circumstances, unavoidable. But they are by no means wholly so, and the perception of this by the individual citizen would mean a great gain to the public health.

* "Maladies Caused by the Air we Breathe Inside and Outside the Home." By Thomas Oliver, M.D., LL.D., F.R.C.P. (London: Baillière, Tindall, and Cox. 1906).

